# Perspectives of lipid metabolism reprogramming in head and neck squamous cell carcinoma: An overview

**DOI:** 10.3389/fonc.2022.1008361

**Published:** 2022-09-16

**Authors:** Xiangwan Miao, Beilei Wang, Kaili Chen, Rui Ding, Jichang Wu, Yi Pan, Peilin Ji, Bin Ye, Mingliang Xiang

**Affiliations:** ^1^ Department of Otolaryngology & Head and Neck Surgery, Ruijin Hospital, Shanghai Jiao Tong University School of Medicine, Shanghai, China; ^2^ Shanghai Key Laboratory of Translational Medicine on Ear and Nose Diseases, Shanghai, China; ^3^ Ear Institute, Shanghai Jiao Tong University School of Medicine, Shanghai, China

**Keywords:** lipid metabolism reprogramming, lipid catabolism, lipid synthesis, lipid uptake, HNSCCs

## Abstract

Recent studies showed that lipid metabolism reprogramming contributes to tumorigenicity and malignancy by interfering energy production, membrane formation, and signal transduction in cancers. HNSCCs are highly reliant on aerobic glycolysis and glutamine metabolism. However, the mechanisms underlying lipid metabolism reprogramming in HNSCCs remains obscure. The present review summarizes and discusses the “vital” cellular signaling roles of the lipid metabolism reprogramming in HNSCCs. We also address the differences between HNSCCs regions caused by anatomical heterogeneity. We enumerate these recent findings into our current understanding of lipid metabolism reprogramming in HNSCCs and introduce the new and exciting therapeutic implications of targeting the lipid metabolism.

## Introduction

Over 850,000 people are diagnosed with head and neck squamous cell carcinomas (HNSCCs) worldwide, and 440,000 people die of it (1, 2). Although human papillomavirus (HPV)-positive HNSCCs patients have better outcomes with overall survival (OS) rate of 70% (3, 4), patients with stage III–IV disease still suffer from local invasion and therapeutic failure, with a poor prognosis and OS of approximately 40% at 5 years (5). The treatment for HNSCCs is individualized, with either surgery or combined with radiotherapy, chemotherapy, target therapy or immunotherapy, as indicated by the pathological or clinical features and anatomical regions (6). Extensive tissue resection, reconstruction, and side effects of radiotherapy and chemotherapy seriously affect the life quality and survival rate of HNSCCs patients, primarily due to impaired swallowing, speaking and breathing functions (7). Because of inadequate nutrient intake, half of the HNSCCs patients are malnourished and about 80% of them lose weight during treatment (8, 9), whereas some lose up to 20% of body weight (10). After exposure to treatments, several metabolic changes occur because of wound repairing and immune response (7), accompanied with other existed metabolism reprogramming in tumors (11).

It’s well known that HNSCCs are highly reliant on glucose metabolism, known as Warburg effect (12, 13). However, nutritional limitation of the total calorie intake in HNSCCs patients promote cancer cell proliferation (14, 15), indicating that not only glucose metabolism, but also other metabolic processes, such as glutamine and lipid metabolism, are vital. As a newly discovered cancer characteristic (16), studies have found that lipid metabolism is reprogrammed in cancers, too (17). Lipid metabolism could support survival, proliferation, invasion, and metastasis in cancer cells by contributing to membrane formation, energy production and signal transduction, and even mediate drug resistance (18, 19). Due to the rapid proliferation rates and high metabolic energy requirements, cancer cells have tremendous demand of lipids (20, 21). Moreover, a variety of intermediate substrates produced by glucose and glutamine metabolism could participate in lipid metabolism, forming a “shortcut” cycling (22). Thus, lipid metabolism reprogramming plays a “vital” role in HNSCCs.

However, it should be noted that the anatomical HNSCCs regions, especially in the neck and supraclavicular regions, mainly contain brown adipose tissue and beige adipose cells (23), which promote energy consumption and help improve the glucose and lipid metabolic disorders (24). And this could partially explain the marked heterogeneity among different head and neck regions (25), especially nasopharyngeal carcinoma (NPC). In NPC, the most common manifestation is cervical lymph node metastasis, which is riches in brown adipose tissue (23). Distant metastasis occurs in about 20% of NPC cases, and half of them are bone/bone marrow metastasis (26), a region where adipocytes predominate (27). Latest studies found that activated brown adipose tissue can reduce glucose around cancers and inhibit cancer growth (28). Because of these differences in adipose tissue distribution, the mechanisms underlying lipid metabolism in different HNSCCs, and other solid carcinomas may vary.

Up to date, most studies were working on the key enzymes involved in lipid uptake and synthesis in HNSCCs, and the upregulation of these enzymes indicates the therapeutic potentials of lipid uptake and synthesis inhibitors in HNSCCs. In this review, we summarize the current studies working on lipid metabolism enzymes and signal transduction molecules and introduce the advancements for lipid metabolism disruption in HNSCCs. Lipids are composed of fat (triglyceride, TG) and lipoid (phospholipid, cholesterol, and cholesterol esters) and both are involved in lipid uptake, synthesis, storage, and catabolism (20). Thus, this review introduces the reprogramming of lipid metabolism in HNSCCs by FA and cholesterol, which are the main substrate for fat and lipoid.

## Lipid uptake in HNSCCs

### Cholesterol uptake

Cholesterol, which plays a crucial role in membrane structure, is absorbed by intestinal enterocytes (29) and used to synthesize very low-density lipoprotein (VLDL) in the liver (30). VLDL is released into the blood and processed into low-density lipoprotein (LDL), which is taken up by low-density lipoprotein receptors (LDLR) on peripheral cells (31). Nicotine in tobacco can induce an increase in LDLR expression in oral epithelial cells, while smoking is an important risk factor for HNSCCS (32). But the blood cholesterol and LDL levels are significantly decreased in oral carcinoma patients (33, 34). These results indicate that HNSCCs require more cholesterol and LDL than normal cells. Daker et al. also found that Epstein-Barr virus encoded RNA (EBERs) up-regulated LDLR and FA synthase (FASN) in NPC cells (35). Besides, experiments on head and neck cancer (HNC) cell lines revealed that the expression of CD36 and LOX-1, another two LDL membrane receptors, were significantly upregulated after exposure to oxidized LDL (oxLDL) (36), which also suggested that the uptake of cholesterol increased in HNSCCSs. However, lipid metabolism varies according to different tumor microenvironment (TME) and progression stages (37, 38). When oxLDL upregulated CD36 in HNC cell lines, the migration of cancer cells were reduced after oxLDL exposure (36). Thus, the regulation of LDL receptors needs further exploration in order to guide the administration of cholesterol uptake inhibitor. The mechanisms underlying cholesterol efflux proteins, such as LXR or ABCA1, in HNSCCs are still lacking, which worth more attention since they affect the total concentration of cholesterol inside the cells, too.

### FA uptake

FA is another essential molecule involved in lipid biosynthesis and serves as a substrate for energy production metabolism. Mammals produce only a few endogenous FAs, which carry a double bond at δ 9 in the hydrocarbon chain. Other necessary FAs, especially polyunsaturated FAs, need to be obtained from food (20, 39). FA are taken up by simple diffusion through the lipid bilayer or by FA transporters on the membrane (22). The currently known FA transporters include differentiated cluster 36 (CD36, also known as FA translocation enzyme), FA transporter family (FATPs, also known as SLC27), and FA binding proteins on the plasma membrane (also known as FABPs). Abnormal elevation of these three proteins occurs in a variety of cancers (20, 40). Among them, CD36 has been studied most comprehensively in HNSCCs. In oral squamous cell carcinoma (SCC), CD36 upregulation promotes tumor metastasis, while its inhibition leads to complete remission or elimination of lymph node and lung metastases in *in vivo* oral carcinoma models (40). These findings suggest the therapeutical use of CD36 inhibitors in advanced HNSCCs patients. What’s more, CD36 inhibitors could reduce the growth of oral SCC cells and inhibited lipid droplet (LD) formation, tumor progression, and metastasis (41–43). And it is important to know that our daily dietary intake may affect the expression of CD36 as Pascual et al. found that dietary palmitic acid (PA) activated CD36 in oropharyngeal carcinoma and stimulated metastasis of cancer cells, which was promoted by a specialized proregenerative extracellular matrix secreted from cancer-associated Schwann cells (44). Thus, nutritional interventions should be considered together with lipid metabolism inhibitors for cancer treatment. Similar to CD36, Rauch et al. found that FABP protein expression was significantly increased in HNSCCs compared to normal tissues (45). Then, Ohyama et al. further found abnormal expressions of FABP4 and FABP5 in tongue carcinoma, whereas only FABP5 was expressed in normal tongue epithelial cells, which showed a higher expression level in injured and cancer tissues (46). Although few studies have evaluated the role of FA transporter family in HNSCCs, these studies revealed that HNSCCs require more FAs than normal cells. However, the killing efficiency of the FA uptake inhibitors should be researched more specifically, along with the optimal duration of use, usefulness and efficiency of nutritional interventions, and long-term side effects.

## Lipid synthesis and storage in HNSCCs

**Figure 1:**
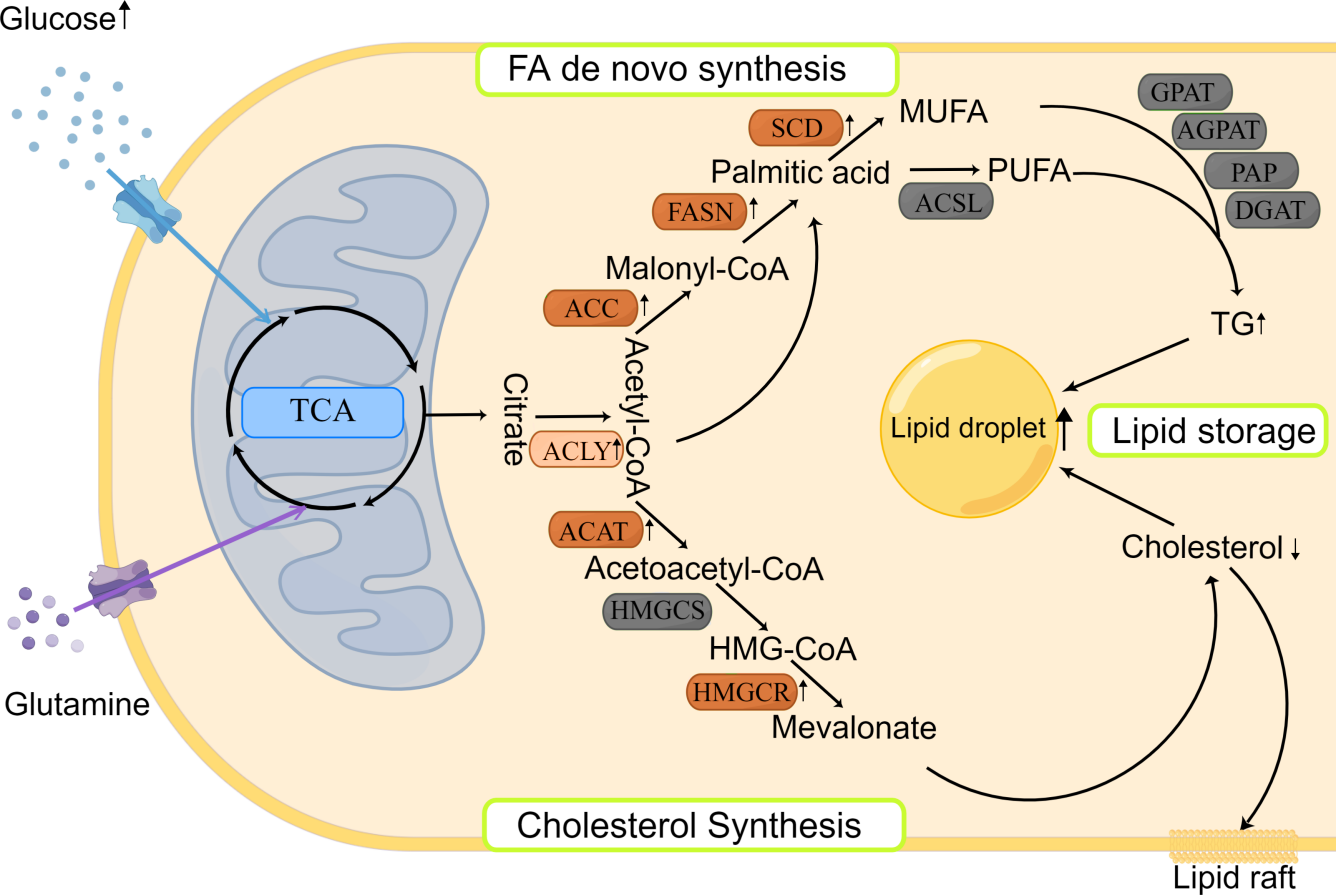
Lipid synthesis and storage in HNSCCs. Lipid synthesis begins with citric acid, produced from the TCA cycle, which is used to synthesize different lipids in the cytoplasm. There are two main pathways involved: FA *de novo* synthesis and cholesterol synthesis. The produced lipids are stored as LDs. Most enzymes involved in lipid synthesis are upregulated in HNSCCs.

Citric acid, produced by the citric acid cycle or glutamine metabolism, is the starting molecule involved in intracellular lipid synthesis. ATP-citric acid lyase (ACLY) converts citric acid to acetyl-CoA and oxaloacetate, which are used to synthesize different lipids in the cells (**Figure 1**). Although there is no direct evidence of ACLY expression in HNSCCs, Zheng et al. found that the long non-coding RNA TINCR could bind to ACLY and protect it from degradation in NPC, which maintained the total acetyl-CoA level in cells (47). In addition, Sur et al. reported that bitter gourd extract could significantly reduce the expression of ACLY, acetyl-CoA carboxylase (ACC), and FASN genes in oral carcinoma, and promote cell apoptosis (48). These results suggest that the ACLY expression is increased in HNSCCs, which may contribute to the survival of cancer cells and ACLY inhibition may be used as a new anticancer treatment in HNSCCs.

### Cholesterol synthesis

Cholesterol biosynthesis begins with the conversion of two molecules of acetyl-CoA to acetoacetyl-CoA by acetyl-CoA acetyltransferase (ACAT). Subsequently, a third acetyl-CoA molecule is synthesized into HMG-CoA by HMG-CoA synthase (HMGCS). HMG-COA reductase (HMGCR) is the next rate-limiting step in cholesterol synthesis and produces mevalonate (**Figure 1**). Mevalonate can be modified to produce different cholesterols with various physiological functions, such as lipid raft in cell membrane (29). Using genetic variation assessment, Gormley et al. reported that there was limited evidence regarding LDL reduction by HMGCR, Niemann-Pick type C1-like 1 (NPC1L1), CETP, or other circulating lipid trait genes on the risk of oral or oropharyngeal carcinoma (49). However, ACAT1 was reported to be associated with poor prognosis of oral SCC (50). This may be explained by the lack of consideration of cholesterol efflux in the previous study, which affects the total quantity of cholesterol inside the cancer cells. Although previous findings related to cholesterol synthesis are controversial and there are limited reports about the expression and prognostic role of cholesterol synthesis-related enzymes in HNSCCs, statins, which are the cholesterol-lowering drugs that act by HMGCR inhibition (51), could induce apoptosis of cancer cells by consuming non-steroidal mevalanoic acid metabolites in HNSCCs (52). Furthermore, statins could enhance the effects of cisplatin with concomitant use and potentiate the efficacy of immunotherapy in HNSCCs (53). These results highlight the potential therapeutic use of statins in HNSCCs, which should be further studied to clarify the mechanisms behind.

### FA *de novo* synthesis

FA *de novo* synthesis begins with the conversion of acetyl-CoA to malonyl-CoA by ACC. Then, acetyl-CoA and malonyl-CoA are catalyzed by FASN to form palmitate, which is further modified by elongase of very long chain fatty acids (ELOVL) enzymes to elongate the length of FA chains. Finally, polysaturated FAs, such as palmitic acid, are desaturated to produce unsaturated FAs by stearoyl-CoA desaturase (SCD) and/or other fatty acyl-CoA desaturases (**Figure 1**). The expression of various rate-limiting enzymes involved in FA *de novo* synthesis was increased in HNSCCs. In HNSCCs with lymph node metastasis, highly phosphorylated ACC expression was found to be associated with poor survival outcomes (54). And ACC2 serves as a vital prognostic indicator and potential therapeutic target in HNSCCs (55). As another key rate-limiting enzyme in FA synthesis, FASN expression was found to be increased in HNSCCs, too. Epstein-Barr virus could promote FASN expression in NPC cells (35, 56) and FASN transcription was increased in cisplatin-resistant SCCs and played a role in cisplatin resistance (57). Furthermore, FASN siRNA inhibited the growth of *in vivo* oral SCC and lymph node metastasis (58), and FASN inhibitors increased the sensitivity to radiotherapy (59). The aforementioned results suggest that inhibition of FA synthesis would be a novel and exciting treatment for HNSCCs. Clinical trials evaluating the efficiency of the FASN inhibitors are currently ongoing on variety of cancers, including oral cancers (NCT02223247) (www.clinicaltrails.gov). Besides, SCD inhibitors could hinder cancer cell proliferation and invasion in oral carcinoma (60, 61), but need more in-depth and long-term studies.

### Total lipid synthesis and storage

After synthesis, FAs bind to different backbones to produce different classes of fat in the body, such as phospholipids and TGs with glycerol is the most common backbone, except phospholipids. FAs produce TGs through several enzymes, including Gly3P phosphate acyltransferase (GPAT), 1-acyl-sn-Gly3P acyltransferase (AGPAT), PA phosphatase (PAP), and DAG acyltransferase (DGAT). TGs are then encapsulated in LDs, which is the main storage form of lipids (**Figure 1**). LD accumulation serves as a phenotype for metastasis initiation, energy storage, and regulatory mechanism of reactive oxygen species in carcinomas (62). HNSCCs show increased LD accumulation, too (63, 64). However, the distribution and mechanism of key enzymes and molecules, such as lipins, in HNSCCs have not been reported previously, and merit further exploration.

## Lipid catabolism in HNSCCs

### Lipolysis

**Figure 2:**
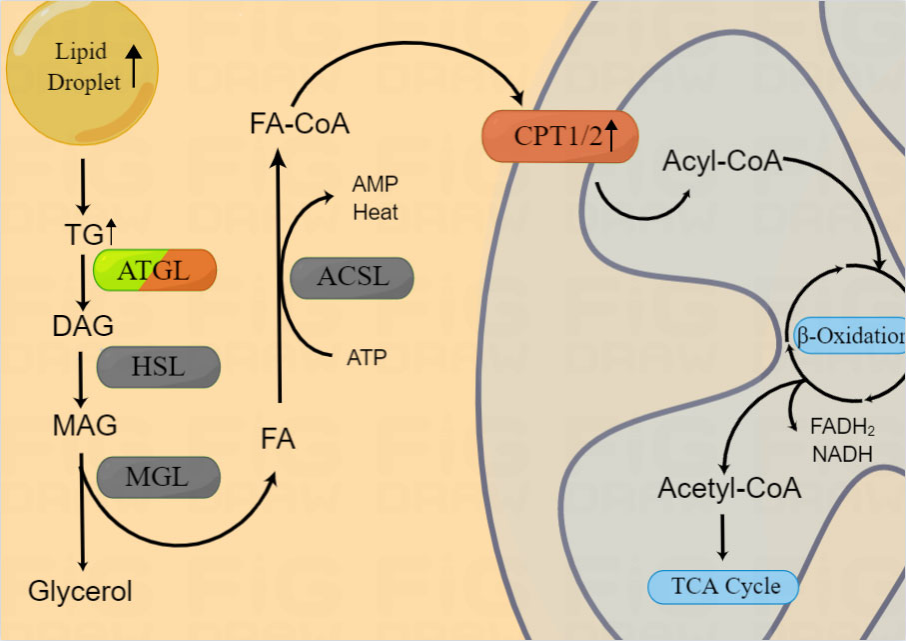
Lipid catabolism in HNSCCs. Enzymes involved in lipid catabolism are shown in the figure. After release from the LD, triacylglycerol is broken into FA-CoA by lipolysis-related enzymes and catabolized by FAO to produce energy and substrates for the mitochondrial TCA cycle. β-oxidization are reported to be upregulated in HNSCCs, however, the expression of ATGL in HNSCCs is still controversial.

In response to the requirements of rapid growth and invasion, intracellular lipolytic enzyme activity is also increased (65). In mitochondria, long chain FAs are transformed into acetyl-CoA through lipid catabolism (20), thereby providing ATP and substrates for lipid synthesis (66). The initial step of lipolysis is the hydrolysis of TG into diacylglycerol (DAG) by lipases. Two main lipases are involved in this process, namely, hormone-sensitive lipase (HSL) and fatty triglyceride lipase (ATGL, also known as phospholipase A2, PNPLA2, or PLA2). Rather than TG, HSL hydrolyzes DAG to monoacylglycerol (MAG), while ATGL almost completely hydrolyzes TGs to release DAG (67, 68). DAG is derived from TGs *via* ATGL, and DAG is hydrolyzed by HSL to 2-MAG. Then, 2-MAG is hydrolyzed by MAG lipase (MGL) to free FAs and glycerol, which is then secreted extracellularly (22) (**Figure 2**).

In 2012, Tripathi et al. found that, along with the Warburg effect, the phosphatidylcholin/lysophosphatidylcholine and phosphatidylcholine/glycerophosphatidylcholine ratios were significantly increased and the activity of ATGL in HNSCCs (oral, tongue, and larynx) was enhanced (69). However, Zhou et al. found that in NPC, ATGL expression was inhibited, lipolysis was reduced, and LD accumulation was increased (63). In addition, they found that low ATGL expression was associated with poor prognosis of patients and ATGL inhibition was regulated by Epstein-Barr virus-encoded membrane latent protein 2A (LMP2A). LMP2A not only promoted lipid accumulation by inhibiting ATGL, but also enhanced migration *in vitro* (64). Thus, the expression and mechanisms of ATGL varied according to the anatomical regions of HNSCCs. As we mentioned above, this gene heterogeneity may be related with the different adipose tissue distribution and lipid metabolism in HNSCCs.

### FA catabolism

In addition to being a metabolic intermediate in lipid anabolism, FAs are an important energy source. FAs are catabolized by fatty acid oxidation (FAO), also known as β oxidation. FA-CoA was transformed into FA-carnitine by carnitine palmityl transferase (CPT) and transported from the cytosol across the outer mitochondrial membrane. Within the mitochondria, FAs are repeatedly cleaved to produce acetyl-CoA, which is recycled into the citric acid cycle to produce the reductive equivalent of oxidative phosphorylation (**Figure 2**). Du et al. found that increased CPT1A-mediated FAO was significantly associated with radiotherapy resistance in NPC (70), suggesting the potential use of combination treatment of FAO inhibitors and radiotherapy. However, the mechanism underlying the role of CPT1/2 in other HNSCCs as well as its other functions require further evaluation.

## Regulation pathways of lipid metabolism reprogramming in HNSCCs

In addition to the key lipid metabolic steps mentioned previously, there are also many important signaling pathways involved in lipid metabolism regulation, such as PI3K/AKT, mTOR, and AMPK pathways, which have been discussed previously and are not included in this review (22, 71). In addition to the above pathways, there is a star lipid regulation pathway, which is involved in the regulation of synthesis of multiple lipids, namely, INSIG/SCAP/SREBPs, and requires further attention. The INSIG/SCAP/SREBPs complex is located on the endoplasmic reticulum but does not have any regulatory activity. After cholesterol or glucose stimulates INSIG, the SCAP/SREBPs complex is transported to the Golgi and cleaved into the activated form. Then, SREBP-1c is released into the cell nucleus and regulates the downstream genes as a transcriptional factor. SREBPs has three main forms, namely, SREBP1a, SREBP1c, and SREBP2. SREBP1 mainly regulates the expression of FA synthesis genes and LDLR, while SREBP2 preferentially regulates the expression of cholesterol biosynthesis genes (20). In NPC, SREBP1 activation mediated lipid synthesis and promoted tumor proliferation and progression (72). However, the distributions, expression levels, and specific mechanisms of INSIG/SCAP/SREBPs in different HNSCCs are still unclear.

## Effects of high-risk factors on lipid metabolism reprogramming in HNSCCs tumor microenvironment

### Tobacco and alcohol

Compared with non-smoker who never drank, those who drank and smoked every day had a 14-fold higher risk for head and neck squamous cell carcinoma (73). Alcohol consumption alone increases the risk for head and neck squamous cell carcinoma (74, 75). Ethanol is oxidized into acetaldehyde after absorption, which forms various proteins and DNA adducts that promote DNA repair failure, lipid peroxidation and metabolism (76). In HNSCCs, there is a significant positive dose-response relationship between prediagnosis alcohol intake and worse OS, especially associated with the fast ADH1B and the slow/nonfunctional ALDH2 genotype combination (77), two dehydrogenase for alcohol and aldehyde. Chronic alcohol exposure decreases the DNA binding ability of PPARα, a nuclear hormone receptor involved in mitochondrial β-oxidation regulation (78, 79), and impairs cholesterol synthesis (80, 81), which may promote cancer progression, and may also occur in head and neck epithelial cells. Another risk factor that HNSCCs patients are frequently exposed to is tobacco. Difference in lipidome signatures can be found between smokers and non-smokers across a number of lipid species (82, 83). Compared with unexposed, active or passive smokers have higher LDL (84–87) and lower HDL (88). Nicotine in tobacco can induce up-regulation of LDLR expression in oral epithelial cells (32). However, the serum levels of total lipids, cholesterol and HDL in patients with oral cancer are significantly reduced, while triglycerides and VLDL are increased (33, 34). Above results support that lipid metabolism reprogramming has a significant relationship with HNSCCs development, although the specific mechanisms of alcohol and tobacco regulation is still unclear.

### Virus infection

As a part of the upper aerodigestive tract, HNSCCs are often affected by viral or bacterial microbes, such as HPV and EBV. Viruses require lipid-mediated endocytosis to enter the cell and HPV proteins L1 and L2 could activate lipid-raft mediated endocytosis to increase its infection (89). The HPV16 E5 protein even can change the lipid composition in cells to help establishing an immune suppressed TME that favors HPV long-term infection (90). HPV16 E6 and E7 could up-regulate lipid synthesis by activating PI3K/AKT/mTOR (91) and SREBPs lipid synthesis signaling pathways (92–94). HPV-positive HNSCCs patients had higher levels of gene expression in TCA cycle, oxidative phosphorylation and β-oxidation, compared with HPV-negative patients (95). However, due to the different adipose tissue distribution, the lipid metabolism regulated by HPV may also varied in different cancers. For example, although HPV is involved in the regulation of cell metabolism in both cervical cancer and HNSCCs, its functions varied. In HPV-associated HNSCCs, it mainly promotes oxidative phosphorylation to obtain energy (96, 97), while in cervical cancer, HPV E6 protein up-regulates lipolysis and down-regulates oxidative phosphorylation (98).

Another well-known virus risk factor in HNSCCs is Epstein-Barr virus, which is also involved in lipid metabolism reprogramming in HNSCCs TME. EBV encoded LMP1 has been reported to regulate glycolysis and lipogenesis in NPC (56, 99, 100). EBV-mediated reprogramming of lipid biosynthesis promotes B-cell activation and differentiation surrounding TME (101, 102), which help shaping a tumor favored TME. At the same time, EBV can also release inflammatory factors, such as IL6, IL-10 and leptin, which promote fat consumption (103–105) and help cancer cells to evade immune surveillance (106). Therefore, virus associated HNSCCs show differences in lipid metabolism compared with non-infectious HNSCCs, which worth more study in the future.

### Dietary interventions

Dietary interventions alter the metabolic substrates concentrations in the TME, which will reprogram the cancer cell metabolism and induce cancer development and progression (107–111). Caloric restriction inhibits the growth of pancreatic cancers and helps limit cancer progression (112). However, the total calory intake restriction intervention does not improve the survival prognosis in HNSCCs, but improves the cancer cells proliferation (14, 15). Whether a hypoglycemic diet will inhibit cancer growth may be determined by the mismatch between the fatty acid desaturation degree and the available specific fatty acid types in the cancer (112). Therefore, the role of lipid metabolism in HNSCCs deserves further investigation.

Ferroptosis is an iron-mediated lipid peroxidation that causes non-apoptotic cell death, which is associated with cancer development and therapy response. Inhibition of GPX4, an important ferroptosis regulation molecule, can sensitize drug-resistant cancer cells in HNSCCs (113). During ferroptosis, polyunsaturated fatty acids (PUFAs) are most susceptible to peroxidation, which can cause the destruction of the lipid bilayer and affect membrane function (114). In oral cancer, glutathione can regulate lipid oxidation by binding to PTGS2 which promotes ferroptosis (115). What’s more, high fat-soluble vitamins, such as Vitamin D is associated with lower risk of cancer (116). Thus essential nutrients such as glutathione (GSH), fat-soluble vitamins A, D and K, which help remove ROS (98) and regulate lipid peroxidation and ferroptosis (117), have potential anticancer application in HNSCCs by promoting ferroptosis.

In addition to essential nutrients, there are many exogenous lipid nutrients with potential cancer killing effects in HNSCCs. Reports have shown that docosahexaenoic acid, a ω-3 fatty acid, can induce the degradation of HPV E6/E7 oncoprotein and promote apoptosis (118). Ergosterol Peroxide extracted from mushroom can increase radiotherapy sensitization in cervical cancer cells (119). Salvianolic acid B extracted from salvia miltiorrhiza, which could can also inhibit the malignant transformation of oral premalignant lesion (120), which has been reported had the protective effect on metabolic homeostasis by regulating PPARγ, FASN, SCD1 and CD36 (121). These results suggest that exogenous unsaturated fatty acids and lipid nutrients extracted from plants may have therapeutical potential in HNSCCs.

## Regulation of lipid metabolism in HNSCCs by cancer associated cells in TME

Apart from cancer cells, cancer-associated cells in the TME also play an important role in the occurrence and development in cancers. Among them, the one that has been studied the most is cancer-associated fibroblasts (CAFs), which can be derived from normal fibroblasts around cancers, mesenchymal stem cells and cancer cells undergoing EMT transformation (27). It interacts with cancer cells and other components in the TME which help forming a tumor-supporting TME (122–124). HPV-negative oropharyngeal cancer cells can stimulate normal fibroblasts to produce HGF and IL-6 (125), and senescent CAFs will secrete more IL-6, COX2 and PGE2 (126). And then, IL-6 further promotes cancer cell invasion, lipid depletion and immunosuppression (103–106). Although there is evidence that CAFs in HNSCCs exhibit similar metabolic characteristics with cancer cells (127, 128), studies on lipid metabolism in CAFs are still lacking. What’s more, Pascual et al. found that dietary PA could induce the cancer associated Schwann cells to secrete a specialized extracellular matrix to promote metastasis (44). All these results support that cancer associated cells help reprogramming the lipid metabolism in HNSCCs TME.

Adipocytes also can differentiate into CAFs (27). Cancer cells could regulate lipid metabolism of adipocytes to produce cancer-associated adipocytes, which are morphologically and functionally different from normal adipocytes (129). Cancer-associated adipocytes then further release fatty acids, mitogens and proinflammatory adipokines to promote the occurrence and development of cancers (130–134). Adipokines such as leptin and adiponectin are lower in HNSCCs patients (135–138), whereas visfatin and chemerinze are higher (28, 139).However, adipocytes distribution in different regions of HNSCCs varies, which may be the reason for the distinct metabolism CAFs subtypes in HNSCCs (128, 140). All these evidences support that cancer associated adipocytes and CAFs play a vital role in lipid metabolism in HNSCCs, but needs more exploration.

## Summary and future prospects

The mask of lipid metabolic reprogramming in HNSCCs is gradually being revealed. Previous studies reported that a variety of lipid metabolic enzymes are upregulated in HNSCCs, but heterogeneous was also existed according to different TME and anatomical regions. Cancer cells are constantly reprogramming their lipid metabolisms in response to the TME and/or metastasis/colonization needs in HNSCCs. In this article, we summarized the previous research on lipid metabolism reprogramming in HNSCCs. However, as shown in **Table 1**, only few lipid metabolism enzymes have been researched and there are still a lot of vacancy in this area which need further exploration in the future. Importantly, HNSCCs comprise a diverse group of cancers that affect the upper aerodigestive tract. The differences in reprogramming of lipid metabolism under different TMEs in HNSCCs require additional studies. Lipid metabolism reprogramming not only shows extensive interaction with other metabolic mechanisms, but also has various crosstalk with surrounding cells, cytokines, growth factors, and even nutrient molecules within the malignant cancer cells. Therefore, the role of lipid metabolism reprogramming in HNSCCs needs additional studies, including, but not limited to, its effects on the immune microenvironment and angiogenesis. Further understanding of the lipid metabolism reprogramming mechanisms, key rate-limiting enzyme functions, and regulatory pathways in HNSCCs may help to develop the potential use of lipid metabolism pathways as targets for anti-tumor therapy, as well as the use of dietary/nutritional interventions to improve the prognosis and life quality of HNSCCs patients.

**Table 1:** Expression of key lipid metabolism enzymes in HNSCCs.

Lipid metabolism type		Enzymes	Expression	Subsites	References
Lipid transportation	Cholesterol uptake	LDLR	Overexpression	Oral epithelial	(32)
				Nasopharyngeal carcinoma	(35)
		CD36	Overexpression	Head and neck carcinoma	(36)
		LOX-1	Overexpression		
	FA uptake	CD36	Overexpression	Oral squamous cell carcinoma	(40)
				Oropharyngeal carcinoma	(44)
		FABPs	Overexpression	Head and neck carcinoma	(45)
				Tongue carcinoma	(46)
Lipid anabolism		ACLY	Overexpression	Nasopharyngeal carcinoma	(47)
				Oral carcinoma	(48)
	Cholesterol synthesis	ACAT1	Overexpression	Oral carcinoma	(50)
		HMGCR	Overexpression	Head and neck carcinoma	(51)
				Head and neck carcinoma	(52)
				Head and neck carcinoma	(53)
	FA *de novo* synthesis	FASN	Overexpression	Nasopharyngeal carcinoma	(35)
				Nasopharyngeal carcinoma	(56)
				Nasopharyngeal carcinoma	(57)
				Oral carcinoma	(58)
				Head and neck carcinoma	(59)
		ACCs	Overexpression	Head and neck carcinoma	(54)
				Head and neck carcinoma	(55)
		SCD	Overexpression	Oral carcinoma	(60)
				Oral carcinoma	(61)
Lipid catabolism	Lipolysis	ATGL	Overexpression	Head and neck carcinoma	(69)
			Low expression	Nasopharyngeal carcinoma	(63)
	FA catabolism	CPT1	Overexpression	Nasopharyngeal carcinoma	(70)

## Author contributions

Conceptualization, XM, BY, and MX; methodology, YP; software, KC and PJ; validation, XM, BW, and BY; investigation, RD; resources, JW; data curation, XM and BW; writing—original draft preparation, XM; writing—review and editing, BY; visualization, BW; supervision, MX; project administration, MX; funding acquisition, XM, BY, and MX. All authors have read and agreed to the published version of the manuscript.

## Funding

The study was supported by grants from the Cultivation Project of the Major Research Plan of the National Natural Science Foundation of China (grant No. 91949119), National Natural Science Foundation of China (No.82101209 and No.82101212), Science and Technology Commission of Shanghai Municipality (grant No.21ZR1440200), the Shanghai Sailing Program (20YF1426400, 19YF1430300), and Ruijin Youth NSFC Cultivation Fund.

## Acknowledgments

Figures were generated by using Figdraw (www.figdraw.com).

## Conflict of interest

The authors declare that the research was conducted in the absence of any commercial or financial relationships that could be construed as a potential conflict of interest.

## Publisher’s note

All claims expressed in this article are solely those of the authors and do not necessarily represent those of their affiliated organizations, or those of the publisher, the editors and the reviewers. Any product that may be evaluated in this article, or claim that may be made by its manufacturer, is not guaranteed or endorsed by the publisher.

## Glossary

**Table:**

ABCA1	ATP Binding Cassette Subfamily A Member 1
ACAT	acetyl-CoA acetyltransferase
ACC	acetyl-CoA carboxylase
ACLY	ATP-citric acid lyase
AGPAT	1-acyl-sn-Gly3P acyltransferase
ATGL	adipose triglyceride lipase
CD36	differentiated cluster 36
CETP	Cholesteryl Ester Transfer Protein
CPT	carnitine palmityl transferase
DAG	diacylglycerol
DGAT	DAG acyltransferase
EBERs	Epstein Barr virus encoded RNA
ELOVL	elongase of very long chain fatty acids
FA	fatty acid
FABPs	FA binding proteins
FA CoA	fatty acyl coenzyme A
FAO	fatty acid oxidation
FASN	fatty acid synthase
FDG-PET	fluorodeoxyglucose-positron emission tomography
GPAT	Gly3P phosphate acyltransferase
HMGCR	HMG-COA reductase
HMGCS	HMG-CoA synthase
HNSCC	Head and neck squamous cell carcinomas
HPV	human papilloma virus
HSL	hormone-sensitive lipase
LD	lipid droplets
LDL	low-density lipoprotein
LDLR	low-density lipoprotein receptors
LMP2A	membrane latent protein 2A
LXR	liver X receptor
MAG	monoacylglycerol
MGL	MAG lipase
NPC	nasopharyngeal carcinoma
NPC1L1	Niemann-Pick type C1-like 1
OS	overall survival
PA	palmitic acid
PAP	PA phosphatase
SCD	stearoyl-CoA desaturase
SUV	standard uptake values
TG	triacylglycerol
TME	tumor microenvironment
VLDL	very low-density lipoprotein
